# Contextualizing new estimates of coding intensity in Medicare advantage and opportunities for further research

**DOI:** 10.1093/haschl/qxag011

**Published:** 2026-01-20

**Authors:** Loren Adler, Yevgeniy Feyman, Benedic Ippolito

**Affiliations:** Center on Health Policy, The Brookings Institution, Washington, DC 20036, United States; National Pharmaceutical Council, Washington, DC 20006, United States; Economic Policy Studies, American Enterprise Institute, Washington, DC 20036, United States

**Keywords:** Medicare advantage, risk adjustment, coding intensity

## Abstract

In this piece, we provide context for new estimates of coding intensity in Medicare advantage (MA) and highlight how researchers can build on them to inform ongoing policy debates. In particular, we identify opportunities to study how insurer responses may affect the intended goals of the Center for Medicare and Medicaid Services’s (CMS) recently introduced risk adjustment model, as well as how potential changes in insurer payments may influence plan design.

The Medicare advantage (MA) program has grown rapidly and become a central focus of health policy debates. Although popular with seniors, critics argue that program costs are higher than they should be, in part because insurers engage in more intense diagnostic coding. In their new paper, Albanese et al.^1^ investigate this fact and argue that recent reforms are likely to mitigate this concern significantly.

MA plans attract enrollees by offering supplemental benefits and lower cost sharing in exchange for network restrictions and utilization management. Despite these features, federal spending per enrollee is higher for beneficiaries who enroll in MA than it would have been if those same people enrolled in traditional Medicare. One reason is that beneficiaries tend to have more clinical diagnoses recorded if they enroll in MA. Greater coding intensity increases enrollees’ “risk scores” and, in turn, payments from the federal government.

In response, CMS recently finalized implementing a new risk adjustment model (commonly referred to as “v28”), which is designed to reduce plans’ ability to increase payments through strategic documentation of diagnoses that are only weakly related to enrollees’ health. For example, the new model eliminated approximately 2000 diagnoses codes identified as particularly susceptible to this type of behavior.

Albanese et al.^1^ apply the v28 model to 2022 data to estimate how large coding differentials would appear had the updated model been in effect, before accounting for behavioral responses of plans and providers. They report that MedPAC-style coding-differential estimates would be roughly halved under v28, and that after accounting the statutory minimum coding adjustment, the remaining “uncorrected” coding impact is approximately 1.5 to 2.0 percentage points. These results suggest that the updated model could substantially reduce the fiscal costs associated with coding pattern differences.

The authors’ analysis is highly relevant to ongoing policy discussions and provides an important benchmark for future research. As they note, their estimates implicitly assume insurer behavior does not change. Thus, their findings likely overstate the model's real-world effect, since insurers and providers may adjust coding strategies in response to new incentives. Future research can compare realized changes in coding intensity to the estimates from Albanese et al.^1^ to assess the extent to which coding responses offset the intended effects of the reform.

If the new risk adjustment model leads to sustained reductions in MA risk scores and payments, it will also create a valuable opportunity to examine how lower reimbursement affects the generosity of benefits (such as prescription drug cost sharing and supplemental benefits)—a topic of perennial interest to lawmakers.

## Supplementary Material

qxag011_Supplementary_Data
